# Mechanisms underlying glucose‐dependent insulinotropic polypeptide and glucagon‐like peptide‐1 secretion

**DOI:** 10.1111/jdi.12478

**Published:** 2016-03-14

**Authors:** Frank Reimann, Fiona M Gribble

**Affiliations:** ^1^ University of Cambridge Metabolic Research Laboratories and MRC Metabolic Diseases Unit WT‐MRC Institute of Metabolic Science Addenbrooke's Hospital Cambridge UK

**Keywords:** Gastric inhibitory polypeptide/glucose‐dependent insulinotropic peptide, Glucagon‐like peptide‐1, Secretion

## Abstract

The incretin hormones, glucose‐dependent insulinotropic peptide and glucagon‐like peptide‐1, are secreted from intestinal K‐ and L cells, respectively, with the former being most abundant in the proximal small intestine, whereas the latter increase in number towards the distal gut. Although an overlap between K‐ and L cells can be observed immunohistochemically or in murine models expressing fluorescent markers under the control of the two hormone promoters, the majority (>80%) of labeled cells seems to produce only one of these hormones. Transcriptomic analysis showed a close relationship between small intestinal K‐ and L cells, and glucose sensing mechanisms appear similar in both cell types with a predominant role of electrogenic glucose uptake through sodium‐coupled glucose transporter 1. Similarly, both cell types produce the long‐chain fatty acid sensing G‐protein‐coupled receptors, FFAR1 (GPR40) and FFAR4 (GPR120), but differ in the expression/functionality of other lipid sensing receptors. GPR119 and FFAR2/3, for example, have clearly documented roles in glucagon‐like peptide‐1 secretion, whereas agonists for the endocannabinoid receptor type 1 have been found to show largely selective inhibition of glucose‐dependent insulinotropic peptide secretion. In conclusion, although K‐ and L cell populations overlap and share key molecular nutrient‐sensing mechanisms, subtle differences between the responsiveness of the different cell types might be exploited to differentially modulate glucose‐dependent insulinotropic peptide or glucagon‐like peptide‐1 secretion.

## Introduction

Glucose‐dependent insulinotropic polypeptide (GIP) and glucagon‐like peptide‐1 (GLP‐1) are gut hormones secreted from specialized enteroendocrine cells within the intestinal epithelium. They are released postprandially and act as circulating markers of food consumption, enabling the body to respond appropriately to food‐derived elevations of blood nutrient concentrations. This is crucial for the control of blood glucose concentrations, as costimulation of pancreatic β‐cells by GIP and GLP‐1 approximately doubles the amount of insulin released in response to an elevation in ambient (blood) glucose concentrations. GLP‐1 and GIP are hence often termed ‘incretins,’ and underlie the ‘incretin effect’ – a well‐documented observation that intravenous glucose infusion at a rate that simulates postprandial blood glucose levels triggers only about half as much insulin release as a matched oral glucose challenge. After the discovery that the insulinotropic effect of GLP‐1 is preserved in most people with type 2 diabetes^1^, GLP‐1 mimetics and inhibitors of GLP‐1 degradation by dipeptidyl peptidase 4 have been developed and licensed for the treatment of type 2 diabetes^2^.

Although a wealth of evidence supports the idea that both GLP‐1 and GIP underlie the incretin effect, there are important differences in the activity and plasma profiles of the two hormones. GIP, for example, stimulates glucagon secretion from pancreatic α‐cells, whereas GLP‐1 inhibits α‐cell activity. GLP‐1, in contrast, has anorexigenic properties, whereas GIP seems to have no effects on food intake. GIP is instead considered pro‐adipogenic, based on several observations, including that knockout of the GIP‐receptor^3^ or immunoneutralization of circulating GIP^4^ are protective against diet‐induced obesity in rodents. As the incretin action of GIP seems to be impaired in patients with type 2 diabetes^1^, it might be postulated that a stimulation of GLP‐1 and an inhibition of GIP secretion could be a therapeutic objective in overweight patients with type 2 diabetes. Further understanding the differences between these two hormones should enable a more targeted approach to their exploitation for the treatment of diabetes and obesity. The present review will focus on the physiology of the enteroendocrine cells secreting these two hormones, the GIP‐expressing K cells and the proglucagon/GLP‐1‐expressing L cells.

## Location: overlapping but distinctive populations of K‐ and L cells

**Figure 1:**
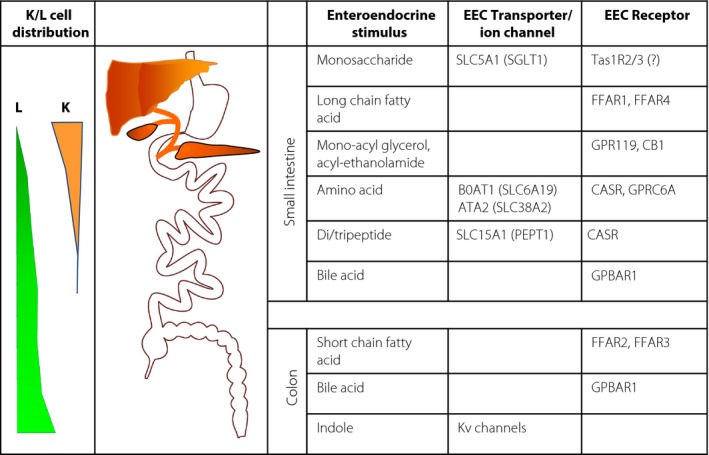
L‐ and K cell distribution and stimulus detection machinery. The majority of K cells are more proximally located than L cells. Fasting and postprandial glucose‐dependent insulinotropic polypeptide (K cells) and glucagon‐like peptide‐1 (L cells) secretion likely reflect the dynamic gradient of different intestinal stimuli along the gut. Physiological activation of the L‐ and K cell detection pathways is shown, involving transporters/ion channels (linked to altered cellular electrical activity) and G‐protein‐coupled receptors, differing between the small intestine and colon. ATA2, amino acid transporter A2 (solute carrier [Slc] Slc38A2); BOAT1, system B(0) neutral amino acid transporter AT1 (Slc6A19); CASR, calcium‐sensing receptor; CB1, cannabinoid receptor 1; EEC, enteroendocrine cell; FFAR, free fatty‐acid receptor; GPBAR1, G‐protein coupled bile‐acid receptor 1; GPR119, G‐protein coupled receptor 119; GPRC6A, G‐protein coupled receptor classC 6A; Kv‐channels, voltage gated potassium channels; SGLT1, sodium‐coupled glucose transporter 1 (Slc5A1); Tas1R, taste receptor type 1.

GIP‐containing cells are found at highest density in the duodenum in a number of species^5^, ^6^, ^7^. GLP‐1‐containing cells can also be found in the proximal small intestine, but increase in number towards the distal small intestine, and in contrast to K cells are also numerous in the colon and rectum (Figure 1). Based on immunohistochemical staining, so‐called LK cells have been described, which were immune‐positive for both GIP and GLP‐1^8^, ^9^. In transgenic mice expressing fluorescent markers under the control of either the GIP or the proglucagon promoter, cells producing the ‘other’ incretin have also been observed; that is, GIP in cells labeled by the proglucagon promoter, and GLP‐1 in cells labeled by the GIP promoter^10^; however, these double‐positive cells only amounted to ~10–20% of the total K‐ and L cell population. Overall secretory responses from cell populations *in vivo* or *in vitro* are thus likely to be dominated by the remaining ~80% of single‐positive cells that produced only GLP‐1 or GIP^6^, and as we describe below, selective stimulation or inhibition of either GIP or GLP‐1 secretion is therefore possible. Nevertheless, the observation that K‐ and L cells additionally produce other hormones, such as CCK^10^, challenges the traditional classification of enteroendocrine cells according to their expression of one (or sometimes two) specific hormones, and suggests a more plastic expression profile that could be affected by external factors, such as the recent exposure of the intestine to nutrients and other luminal stimulants. Given the relatively rapid turnover of enteroendocrine cells in the small intestine every ~5 days^11^, it seems plausible that recent nutritional availability could result in changes to the overall enteroendocrine cell population within days or weeks. In a recent study to identify the effects of a high‐fat diet on mouse L cells, however, we observed a general downregulation of many enteroendocrine cell‐specific genes rather than a switch to the preferential production of an alternative hormone^12^.

## Glucose sensing: similar mechanisms operate in both K‐ and L cells

Given the importance of both GIP and GLP‐1 for the incretin effect, one of the most investigated secretory stimuli of gut hormone secretion is glucose. Both K‐ and L cells in mixed primary intestinal epithelial cultures failed to respond to glucose when the sodium‐coupled glucose transporter 1 (SGLT1) was inhibited either pharmacologically or genetically^13^, ^14^, ^15^, ^16^. Indeed, a wealth of *in vitro* and *in vivo* data have suggested that the rapid elevations in plasma GIP and GLP‐1 concentrations after glucose ingestion are directly linked to the electrogenic uptake of glucose by K‐ and L cells, resulting in membrane depolarization, voltage‐gated calcium entry and enhanced rates of vesicular exocytosis^17^. More extensive phenotyping of global SGLT1 knockout mice, however, showed differences between the release patterns of GIP and GLP‐1, which are likely related to the different locations of K‐ and L cells along the gastrointestinal tract axis^18^. In this mouse model, the GIP response to an oral glucose tolerance test was abolished, consistent with the proposed SGLT1‐dependent coupling of glucose absorption to GIP secretion in K cells. By contrast, whereas the early GLP‐1 response ~5 min after a glucose gavage was abrogated in SLGT1 knockout mice or in mice treated with an SGLT1 inhibitor^13^, ^18^, elevated plasma GLP‐1 concentrations were observed at later time‐points^18^. The findings support the idea that the rapid secretion of GLP‐1 and GIP from the proximal small intestine after a glucose load is linked to SGLT1‐dependent glucose absorption, but suggest that alternative sensory mechanisms operate in the distal gut. Inhibition of glucose absorption in the upper gastrointestinal tract in SGLT1 knockout mice results in a dramatic increase in glucose delivery to the distal gut with its higher density of L cells^18^, likely underpinning the delayed elevation of GLP‐1 levels in these mice. The mechanism by which this glucose load is sensed by the distal ileum and/or colon remains unclear. Candidate pathways include the bacterial fermentation of distally‐delivered carbohydrate to metabolites, such as short chain fatty acids that are then sensed by G‐protein‐coupled receptors, such as GPR43^19^, or metabolism of the sugar within L cells resulting in the activation of an alternative downstream signaling pathway. Neither of these hypotheses has yet been validated experimentally.

Whereas the SGLT1‐dependent pathway is common to K‐ and L cells, we observed some differences between the responsiveness of the glucose‐sensing machinery underlying GIP and GLP‐1 secretion from small intestinal primary murine epithelial cultures. Whereas α‐methyl‐glucopyranoside, a non‐metabolizable SGLT1 substrate, enhanced GLP‐1 secretion in the absence of other stimuli^16^, it only became an effective GIP secretagogue at elevated cyclic adenosine monophosphate (cAMP) levels^14^. Interestingly, the responsiveness to tolbutamide, an inhibitor of adenosine triphosphate‐sensitive potassium (K_ATP_) channels, showed the reverse dependence, triggering GIP responses in the absence, but not presence, of the cAMP‐raising agents, forskolin and 3‐isobutyl‐1‐methylxanthine (IBMX)^14^. K_ATP_ channel closure has itself been postulated as a mechanism underlying K‐ and L cell glucose sensing, similar to its well‐established role in mediating glucose‐dependent insulin secretion from pancreatic β‐cells. In support of this idea, K_ATP_ channel inhibition enhanced GIP and GLP‐1 secretion from intestinal cultures, triggered GLP‐1 secretion from the enteroendocrine cell line, GLUTag^20^, and increased GLP‐1 release from perfused rat intestine^21^. However, there is little data supporting the idea that K_ATP_ channel closure triggers gut hormone secretion in an intact animal. Mice lacking the K_ATP_ channel subunit Kir6.2 did not show impaired glucose‐triggered gut hormone secretion^22^, ^23^; and in humans, the K_ATP_ channel inhibitor, glibenclamide, had no effects on GIP and GLP‐1 concentrations before or during an oral glucose tolerance test^24^. K_ATP_‐channel closure has, however, been suggested to underlie fructose‐stimulated GLP‐1 secretion^25^. Fructose is not a substrate for sodium‐coupled monosaccharide transport, and instead enters cells through the facilitative transporter GLUT5. Interestingly, fructose does not stimulate GIP secretion in healthy rodents and humans^25^, but GIP release in response to fructose has been reported in diabetic mouse models^22^, ^26^. However, opening of K_ATP_‐channels with diazoxide did not abolish fructose‐stimulated GIP secretion in diabetic mice, and even the GLP‐1 response to oral fructose remained intact in mice lacking the K_ATP_ channel subunit, Kir6.2^23^. In view of the widespread agreement that K_ATP_ channels are expressed and functional in K‐ and L cells, the results unanimously support the idea that the resting K_ATP_ conductance is already very small in native K‐ and L cells *in vivo*, and that any differences observed between *in vivo* and *in vitro* experiments might reflect differences in the metabolic status of the enteroendocrine cells under the different conditions.

Another physiological glucose‐sensing mechanism involves the G‐protein‐coupled receptor heterodimer of TAS1R2 and TAS1R3, that underlies sweet taste sensation in the tongue. Impaired postprandial GLP‐1 levels have been observed in mice lacking α‐gustducin, a component of the downstream taste receptor signaling pathway^27^, and both GIP and GLP‐1 secretion have been reported from the GLUTag cell line in response to high concentrations of artificial sweeteners^28^. We, however, were unable to show a role for TAS1R2/3 in hormone secretion from murine K‐ or L cells in primary culture^14^, ^16^, and artificial sweeteners failed to elevate plasma incretin hormone levels in human volunteers^29^. Other studies also failed to show artificial sweetener‐stimulated GIP secretion in mice *in vivo* 22 or GLP‐1 secretion in a perfused rat intestinal preparation^21^, questioning the importance of TASR1R2/3 in incretin secreting cells.

## G‐protein‐coupled receptors: candidates for selectively targeting K‐ and L cells

The development of transgenic mice with fluorescently tagged K‐ or L cells has enabled the transcriptomic analysis of these different cell types. Similarities and differences between K‐ and L cell populations were observed for the expression of a number of G‐protein‐coupled receptors. Both K‐ and L cell populations, for example, were found to express messenger ribonucleic acids (mRNAs) encoding the free‐fatty acid receptors, FFAR1 (GPR40) and FFAR4 (GPR120)^14^, ^16^. GPR40 activation has, for example, been linked to the stimulation of GLP‐1 secretion in experiments using *Gpr40* knockout mice^30^, as well as by the application of GPR40 agonists to the perfused rat intestine^31^. Recent data, by contrast, convincingly showed a reduction in lard‐triggered GIP‐responses in mice lacking *Gpr120* 32. In primary intestinal cultures from mice lacking either *Gpr40* or *Gpr120,* however, we observed diminished secretion of both GIP and GLP‐1 in response to oleate (Reimann and Gribble unpublished observation). Thus, although the data linking GPR120 activation to GIP secretion and GPR40 activation to GLP‐1 release are robust, it is currently unclear whether either of these receptors plays a relatively greater role in any particular enteroendocrine cell type.

Other lipid‐derived stimulants, such as mono‐acyl‐glycerol, activate GPR119, mRNA for which is expressed in both K‐ and L cells^14^, ^16^. Whereas a small molecule GPR119 agonist elevated GLP‐1 and GIP concentrations in mice^33^, however, the natural GPR119 ligand oleoylethanolamide (OEA) was not a good stimulus of GIP‐secretion from primary epithelial cultures^14^. GLP‐1 secretion, by contrast, was stimulated by OEA from both small and large intestinal‐derived murine cultures^34^. In the colon, this was mediated by GPR119, as shown by the loss of OEA‐triggered GLP‐1 release in colonic cultures from mice lacking GPR119 specifically in proglucagon expressing cells. Similar experiments carried out in small intestinal cultures from these mice, however, showed that the OEA‐triggered GLP‐1 secretory response in the upper gastrointestinal tract was not GPR119‐dependent^34^, suggesting that OEA recruits alternative mechanisms in duodenal/jejunal L cells, and that this pathway might not be sufficiently active in K cells to trigger GIP secretion.

The related compounds, mono‐arachidonoyl‐glycerol and arachidonoylethanolamine (anandamide), are agonists for the endocannabinoid receptor, CB1 (encoded by the gene *Cnr1*), which is predominantly G_i_‐coupled. *Cnr1* is highly expressed in small intestinal K‐ and L cells, with a tendency for higher expression in K‐ than L cells, but was not detected in L cells from the colon^35^. Consistent with the known G_i_ coupling of CB1, anandamide was shown to inhibit IBMX‐triggered GIP secretion *in vivo*, an effect that was blocked by the CB1‐antagonist AM251. Interestingly, GLP‐1 secretion from the same cultures was not affected by anandamide, despite the relatively high expression of *Cnr1* in small intestinal L cells. Pretreatment of mice with anandamide delayed GIP, but not GLP‐1 responses to an oral glucose tolerance test^35^. These findings raise questions about why the same receptor is apparently more effectively coupled to inhibition of secretion in K‐ than L cells, despite relatively high expression levels in both.

Ligands for other predominantly G_i_‐coupled receptors expressed in both K‐ and L cells, by contrast, tend to have similar effects on both GIP and GLP‐1 secretion. Somatostatin strongly suppressed the elevation of cAMP triggered by forskolin and/or IBMX in both cell types, and the effect was at least partly mediated through SSTR5^35^. The most likely sources of somatostatin are nearby intestinal D cells, this being an example of how paracrine signals can integrate responses to luminal signals within the epithelium. Galanin, most likely secreted from enteric neurons, similarly recruits GALR1 in both K‐ and L cells, resulting in a G_i_‐dependent suppression of cAMP levels^36^, and exemplifying that enteroendocrine cells likely integrate responses to luminal nutrients with signals arriving through the enteric nervous system. G_i_‐coupled receptors, such as GALR1, have been reported to inhibit electrically excitable cells through activation of G‐protein activated inwardly rectifying potassium (GIRK) channels, mediated by the G‐protein βγ‐subunit. Interestingly, whereas both K‐ and L cells showed enriched expression of mRNAs encoding GIRK channels, and although GIRK‐inhibition had no effect on the ability of galanin to inhibit GLP‐1 secretion, only GLP‐1, but not GIP, secretion could be inhibited by co‐application of the GIRK‐activator, ML297, with IBMX or glucose^36^. This might point to a difference in the role of potassium conductances in the stimulus secretion coupling of K‐ and L cells, but further work assessing possible differences in the electrical activity of these enteroendocrine cell types and its relationship to hormone secretion is required.

Given the calcium dependence of a number of proteins involved in the exocytotic pathway, predominantly G_q_‐coupled receptors should be good targets to stimulate incretin secretion. The similar expression levels in K‐ and L cells of mRNAs encoding the G_q_‐coupled receptor, FFAR1, have been mentioned above. The related short‐chain fatty acid receptor FFAR2 (GPR43) by contrast seems more abundant in L‐ than K cells (Affymetrix chip array probe 1425216 RMA‐values are 2,143 for fluorescently tagged L‐ and 154 for fluorescently tagged K cells isolated from the small intestine, with the latter value being similar to values observed for the non‐fluorescent control cells; Reimann and Gribble unpubl.), supporting the reported importance of GPR43 in short‐chain fatty acid stimulated GLP‐1 secretion^19^. However, whereas fluorescent reporter mice for FFAR2 had only a few labeled enteroendocrine cells, reporter mice for the other short chain fatty acid receptor FFAR3 (GPR41) showed strong labeling of a number of enteroendocrine cells in the small intestine, including K‐ and L cells^37^. A recent publication reported a blunting of GLP‐1, but not GIP, secretion in response to orally‐administered butyrate in *Ffar3* knockout mice^38^, and further work will be required to clarify the relative roles of these receptors in incretin‐secreting cells. Another G_q_‐coupled receptor, presumably underlying modulation of enteroendocrine secretion by the enteric nervous system, as it is activated by neuromedin C and gastrin‐releasing peptide, is the bombesin receptor 2. Bombesin receptor 2 mRNA was found to be selectively enriched in L cells, but not K cells, and, consistent with this finding, bombesin increased calcium concentrations in L cells, but not K cells, and triggered GLP‐1, but not GIP, secretion from primary small epithelial cultures as well as in a perfused intestinal preparation^39^.

Whether any of the predominantly G_q_‐coupled receptors will be good targets for selective stimulation of GLP‐1 secretion *in vivo* will have to await further research. It should be noted, however, that a recent report of a so‐called FFAR1 ‘superagonist,’ capable of triggering robust GLP‐1 secretory responses, stresses the importance of the dual action of such compounds in recruiting both G_q_‐ and G_s_‐coupled pathways^40^. This latter notion is supported by our observation that raising intracellular cAMP levels with forskolin and IBMX boosts GIP and GLP‐1 secretory responses to a number of agents that elevate enteroendocrine cell cytosolic Ca^2+^ concentrations, in primary epithelial cultures^17^. A predominantly G_s_‐coupled receptor expressed in L cells, but not K cells, is the melanocortin receptor 4, and increased GLP‐1 and PYY secretion in response to agonists has been shown^41^, although currently the nature or source of the physiological ligand for this receptor on L cells is uncertain. Other predominantly G_s_‐coupled receptors, expression of which is enriched in L cells, include GPR119 (see above) and the bile acid‐sensitive receptor, GPBAR1 (TGR5). Although there seems to be some expression of *Gpbar1* in K cells, it is not enriched compared with the surrounding cells (Reimann and Gribble unpublished observation), making GPBAR1 one of the more promising receptors to ‘selectively’ stimulate GLP‐1 secretion. However, we recently showed that bile acids need to access the basolateral rather than the apical/luminal surface of L cells to stimulate GLP‐1 secretion through GPBAR1^42^, and a similar observation was reported for agonists of GPR40^31^. As melanocortin receptor 4 was also located to the basolateral side^41^, the interesting question arises if any GPCRs directly sample the luminal contents. If all L cell GPCRs are instead located on the basolateral membrane, the potential hope to develop agents with limited systemic bioavailability to avoid off‐target side‐effects as a result of action on other cells expressing the receptors in question would be unfounded.

## Conclusion

Although the success of GLP‐1 analogs/mimetics in the treatment of type 2 diabetes and the correlation of strongly elevated postprandial GLP‐1 levels after Roux‐Y gastric bypass surgery^43^ strongly suggests benefits of recruiting endogenous GLP‐1 reserves as a not yet exploited treatment alternative, the situation for GIP is less clear. Arguments have been put forward for developing both GIP‐receptor agonists and antagonists^44^. The differences observed in murine K‐ and L cells suggest that it is possible to elevate one incretin preferentially by external stimuli, but further work will be required before translation into a clinical therapy.

## Disclosure

The authors declare no conflict of interest.
